# Application of High-Throughput Next-Generation Sequencing for HLA Typing on Buccal Extracted DNA: Results from over 10,000 Donor Recruitment Samples

**DOI:** 10.1371/journal.pone.0165810

**Published:** 2016-10-31

**Authors:** Yuxin Yin, James H. Lan, David Nguyen, Nicole Valenzuela, Ping Takemura, Yung-Tsi Bolon, Brianna Springer, Katsuyuki Saito, Ying Zheng, Tim Hague, Agnes Pasztor, Gyorgy Horvath, Krisztina Rigo, Elaine F. Reed, Qiuheng Zhang

**Affiliations:** 1 UCLA Immunogenetics Center, Department of Pathology & Laboratory Medicine, Los Angeles, CA, United States of America; 2 University of British Columbia Clinician Investigator Program, Vancouver, BC, Canada; 3 National Marrow Donor Program, Minneapolis, MN, United States of America; 4 One Lambda, Thermo Fisher Scientific, Canoga Park, CA, United States of America; 5 Omixon LTD, Budapest, Hungary; ICELAND

## Abstract

**Background:**

Unambiguous HLA typing is important in hematopoietic stem cell transplantation (HSCT), HLA disease association studies, and solid organ transplantation. However, current molecular typing methods only interrogate the antigen recognition site (ARS) of HLA genes, resulting in many *cis-trans* ambiguities that require additional typing methods to resolve. Here we report high-resolution HLA typing of 10,063 National Marrow Donor Program (NMDP) registry donors using long-range PCR by next generation sequencing (NGS) approach on buccal swab DNA.

**Methods:**

Multiplex long-range PCR primers amplified the full-length of HLA class I genes (A, B, C) from promotor to 3’ UTR. Class II genes (DRB1, DQB1) were amplified from exon 2 through part of exon 4. PCR amplicons were pooled and sheared using Covaris fragmentation. Library preparation was performed using the Illumina TruSeq Nano kit on the Beckman FX automated platform. Each sample was tagged with a unique barcode, followed by 2×250 bp paired-end sequencing on the Illumina MiSeq. HLA typing was assigned using Omixon Twin software that combines two independent computational algorithms to ensure high confidence in allele calling. Consensus sequence and typing results were reported in Histoimmunogenetics Markup Language (HML) format. All homozygous alleles were confirmed by Luminex SSO typing and exon novelties were confirmed by Sanger sequencing.

**Results:**

Using this automated workflow, over 10,063 NMDP registry donors were successfully typed under high-resolution by NGS. Despite known challenges of nucleic acid degradation and low DNA concentration commonly associated with buccal-based specimens, 97.8% of samples were successfully amplified using long-range PCR. Among these, 98.2% were successfully reported by NGS, with an accuracy rate of 99.84% in an independent blind Quality Control audit performed by the NDMP. In this study, NGS-HLA typing identified 23 null alleles (0.023%), 92 rare alleles (0.091%) and 42 exon novelties (0.042%).

**Conclusion:**

Long-range, unambiguous HLA genotyping is achievable on clinical buccal swab-extracted DNA. Importantly, full-length gene sequencing and the ability to curate full sequence data will permit future interrogation of the impact of introns, expanded exons, and other gene regulatory sequences on clinical outcomes in transplantation.

## Introduction

The HLA region contains the most highly polymorphic genes in the human genome. To date, over 10,574 distinct HLA class I alleles and 3,658 class II alleles have been recognized (IMGT/HLA database, December 2015). Although a high degree of HLA polymorphism is important to combat pathogens, it creates a significant barrier for HSCT [1,2] and solid organ transplantation between unrelated individuals [3]. In addition, specific HLA alleles are associated with development of autoimmunity and drug hypersensitivity [4]. Therefore, HLA genotyping is used for HLA matching of donor–recipient pairs in HSCT, identification of humoral responses to donor antigens in solid organ transplantation, and personalized risk assessment of HLA-associated autoimmune diseases and adverse drug reactions.

Over the past two decades, molecular HLA typing techniques have replaced serological methods in clinical applications to provide more precise results. Three basic methods used in conjunction with polymerase chain reaction (PCR) employ sequence-specific oligonucleotide probes (SSOP), sequence-specific primers (SSP), and sequencing-based typing (SBT) [5]. However, due to their low throughput and high cost, HLA typing using these methods principally focuse on the antigen recognition site (ARS) of HLA genes (exon 2 and 3 for HLA class I and exon 2 for class II) where the polymorphisms are predominantly found. Restricting HLA typing to the ARS regions hampers the assignment of high-resolution genotypes and the identification of null alleles with variants outside of the ARS. In addition, these conventional DNA typing methods cannot distinguish between polymorphisms residing on the same chromosome (*cis*) or different chromosomes (*trans*), thereby creating ambiguities that are difficult, time-consuming and expensive to resolve [6]. Recently, a number of groups have successfully leveraged the throughput power of massive parallel sequencing, or NGS, to deliver unambiguous HLA typing with relatively low cost [7–15]. Significant cost reduction is expected with simplified protocols, improved efficiency of reagents/chemistries, and introduction of automated platforms.

Since the initial feasibility study published in 2009 [16,17], several other groups have developed variations in the technique and strategy of HLA genotyping by NGS; however, most of the studies are small in sample size [9,10,12–15,18,19], and the majority were performed using blood samples. Here we describe the development and validation of a high-throughput HLA typing workflow using buccal swab samples in our laboratory [11]. Using this method, we successfully converted our National Marrow Donor Program (NMDP) registry typing contract from traditional typing using the SSO/SBT methodology to high-resolution NGS typing starting October 2014. The NMDP is the largest HSCT donor registry in the world with more than 11 million potential donors listed. Currently, buccal swabs are utilized as a noninvasive sample collection method for NMDP registry donors. However, buccal swab DNA has several limitations that pose unique challenges for typing by NGS. First, buccal-derived DNA yield is much lower than that obtained from peripheral blood. It has been reported that epithelial cells recovered from the mouth are usually superficial and about 25% of these cells are in the process of apoptosis [20]. Second, DNA isolated from cheek cells may contain exogenous DNA from bacteria that interfere with the downstream PCR efficiency and data analysis [20]. Finally, buccal DNA is prone to nucleic acid degradation, which may limit the success of long-range PCR. In this manuscript, we report the development of an automated, NGS-based, HLA typing method for registry typing at HLA-A, -B, -C, -DRB1, -DQB1 on buccal specimens which can overcome these limitations to provide high quality, high-resolution HLA typing.

## Materials and Methods

### Buccal swab (g)DNA samples

Sterile cotton tipped applicator swabs kits (LabCorp, USA) were used to collect buccal epithelial cells by brushing the cotton tip against the inside of mouth cheek for 10 seconds. After receiving buccal swab samples from the NMDP, DNA was isolated using QIAamp 96 DNA Swab BioRobot Kit (Qiagen, Germany) on the Qiagen BioRobot Universal System (Qiagen, Germany) before downstream application. Research approval for performing NGS sequencing on these samples was granted by the UCLA Institutional Review Board (IRB#14–000516).

### PCR amplification, pooling, clean-up

**Fig 1:**
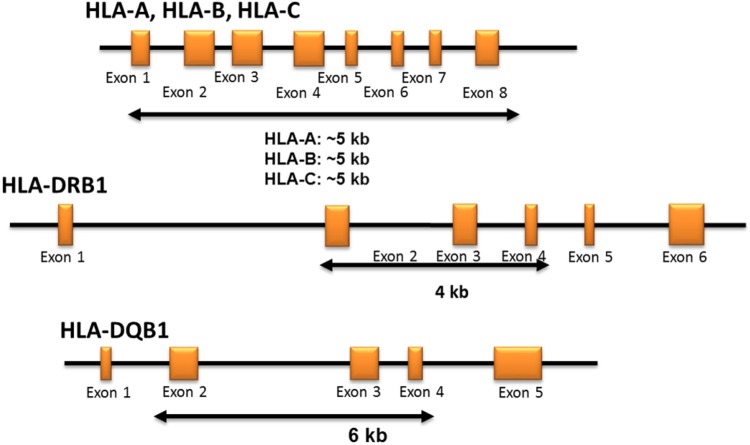
HLA typing strategy. The primer design captures full-length HLA class I genes (HLA-A, -B, -C) and exons 2–4 of DRB1 and DQB1 genes. There are two multiplex primer sets: Class I primer mix includes HLA-A, -B, -C, and Class II primer mix includes DRB1 and DQB1.

Multiplex long-range PCR primers (One Lambda, Canoga Park, CA) were designed to co-amplify HLA-A, -B, -C from promoter to 3’-UTR. For HLA class II genes, long-range PCR primers amplified HLA-DRB1 and DQB1 beginning at exon 2 through part of exon 4 (One Lambda, Canoga Park, CA) in separate reactions (**Fig 1**). In brief, each PCR reaction consisted of: 5μL of buccal swab gDNA, 4μL polymerase buffer, 1.6μL dNTP mixture, 5μL primer mix, and 0.8μL PrimeSTAR GXL DNA polymerase (TaKaRa Bio Inc., Japan), making a final volume of 20μL. GeneAmp PCR system 9700 (Life Technologies, Carlsbad, CA) thermal cycler conditions for class I genes were: 94°C for 2 min, followed by 35 cycles of (98°C for 10 s, 70°C for 3 min). For class II, the optimized conditions were: 94°C for 2 min, followed by 35 cycles of (98°C for 10 s, 69°C for 3 min). PCR products were confirmed on 0.8% agarose gel (Sigma–Aldrich, St. Louis, MO), and then mixed with Agencourt AMPure XP beads (Beckman Coulter, Brea, CA) in 0.6× PCR reaction volume to undergo purification using Biomek NX (Beckman coulter, Brea, CA). Qubit fluorometer 2.0 (Life technologies, Grand Island, NY) was used to quantitate and normalize amplicon concentrations, accounting for the different amplicon fragment lengths; this was followed by equimolar pooling of class I and class II PCR products.

### Library construction

Amplicons of HLA class I and class II genes were sheared using Covaris M220 (Covaris, Woburn, MA). We aimed to obtain an insert size of 500–600 bp to maximize the phasing of linked polymorphisms. Amplicon fragments underwent further purification with Agencourt AMPure XP beads in 1.6× reaction volume. Sheared amplicons then underwent library preparation using the TruSeq Nano DNA sample preparation kit (Illumina, San Diego, CA). Library construction was automated on the Biomek FX (Beckman coulter, Brea, CA). After end-repair, library templates proceeded to double size selection: large fragments were removed using diluted AMPure XP beads in 1.6× reaction volume; small fragments were removed by mixing 30μL of undiluted AMPure XP beads with 250μL of sample supernatant containing the DNA of interest. Following adenylation, Illumina adapter indices were ligated to the purified fragments, such that pooled PCR products from each clinical sample were labeled with a unique adaptor index. From each library sample, 25 μLwas then mixed with 5μL PCR Primer Cocktail and 20μL Enhanced PCR Mix to carry out target library enrichment with a limited-cycle PCR: 95°C for 3 min, followed by 8 cycles of (98°C for 20 s, 60°C for 15 s, 72°C for 30 s), and 72°C for 5 min. Enriched libraries were then purified with AMPure XP beads in a 1:1 ratio.

### Library normalization, pooling, and sequencing on MiSeq

Sequence-ready libraries were validated and quantitated on the High Sensitivity D1000 ScreenTape (Agilent Technologies, Santa Clara, CA) to allow for library normalization and equimolar pooling of all study samples on the Biomek FX (Beckman coulter, Brea, CA). Pooled libraries were diluted and loaded at 12 pM on a MiSeq flow cell, with 5% phiX spiked in. Paired end sequencing runs were performed using MiSeq Reagent Kit v2, 500 cycles (Illumina, San Diego, CA).

### HLA sequence data analysis and genotype calling

Raw sequence outputs were imported as unpacked and gzipped FASTQ files into Omixon Twin V1.0.7 software (Omixon, Budapest, HU) for read alignment and genotype calling (using IMGT/HLA Database 3.19.0 as reference). A read length of 15 bp or greater was the prerequisite for read alignment. The default minimum coverage threshold used to assign genotypes was set at 100.

### Luminex LABType SSO validation

SSO validation was performed using the LABType SSO kit (One Lambda, Inc., USA). 2μL of buccal swab g(DNA) was used for amplification. Next, 5 μL of amplified DNA was denatured at room temperature for 10 min. DNA was hybridized to oligonucleotide probes immobilized on Luminex beads at 60°C for 15 min. After labeling, the tray was loaded on a Luminex 100/200 IS Flow Analyzer (Luminex Corporation, USA) for data generation and analysis using the HLA Fusion software (One Lambda, Inc., USA).

### SBT validation of exon novelty

Variants identified in exon 1–4 of HLA-A, -B, exon 1–7 of HLA-C, and exon 2–3 of HLA-DRB1, -DQB1 were verified using the SBT resolver (Conexio Genomics Ltd, Australia). Mutations residing in other locations were validated using in-house SBT primers. 5μL of buccal swab g(DNA) was used for long-range PCR followed by purification with ExoSAP (Affymetrix, CA). The BigDye Terminator v1.1 Cycle Sequencing (Life technologies, NY) reaction consisted of: Ready Reaction Premix 4μL, BigDye Sequencing Buffer 2μL, Primer (3.2 pmol), purified template 2μL, and H_2_O to make-up a volume of 20μL followed by incubation at 96°C for 1min and 25 cycles of (96°C for 20 s, 50°C for 30s, 60°C for 2min). Sequence products were purified using ethanol precipitation and eluted with 15μL of Hi-Di™ Formamide (Life Technologies, NY) before loading on a 3730xl DNA analyzer (Applied BiosystemsUSA). Data was analyzed using UType.

## Results

### UCLA high-throughput NGS workflow and long range PCR

**Fig 2:**
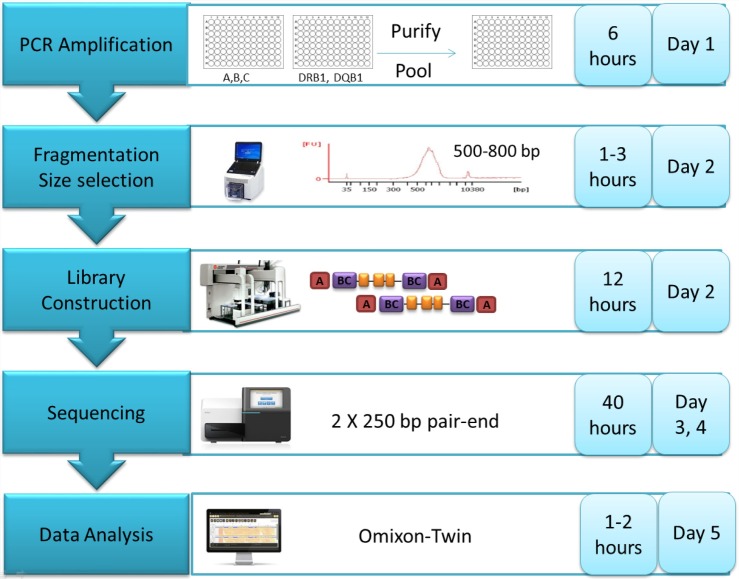
High-throughput NGS workflow. High-throughput NGS workflow begins with multiplex long range PCR of A, B, C and DRB1, DQB1. After PCR, amplicons are purified and pooled in equimolar concentrations. Sheared amplicons then undergo library preparation by using the Illumina TruSeq Nano Kit. To maximize throughput, each clinical sample is labeled with unique dual indices. 2×250 bp paired-end sequence data from the Illumina MiSeq are exported and analyzed using Omixon Twin1.0.7, with 3.19.0 IMGT/HLA database serving as the reference.

Our laboratory developed an automated, high-throughput HLA typing workflow by integrating the Beckman FX robotic liquid handler with the Illumina MiSeq. This method allows for high volume typing at 5 HLA loci (HLA-A, B, C, DRB1 and DQB1) with a rapid 5-day turn-around-time (TAT) to genotype reporting (**Fig 2**). This strategy was able to resolve all third-field ambiguities for HLA class I and all second-field ambiguities for class II alleles. Since DRB1 and DQB1 genes were only partially amplified, ambiguities involving DRB1*04:07/DRB1*04:92 (exon 4), DRB1*09:01/DRB1*09:21 (exon 4), and DRB1*12:01/DRB1*12:10 (exon 1) were still encountered.

**Fig 3:**
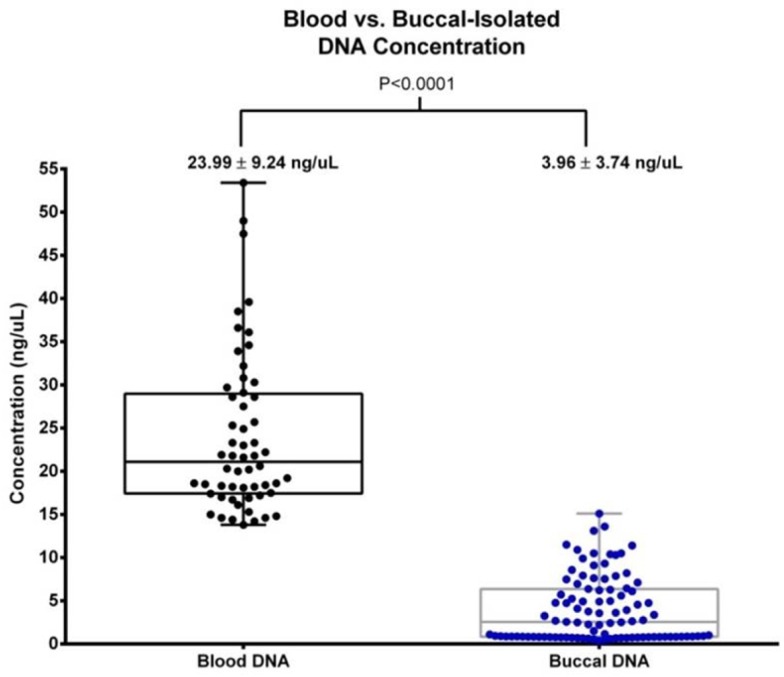
Comparison of DNA concentration in buccal swab and blood samples. The average concentration of buccal-isolated DNA was 3.96 ± 3.74 ng/μL in 100μL volume, which was significantly lower than DNA derived from blood samples (23.99 ± 9.24 ng/μL).

**Fig 4:**
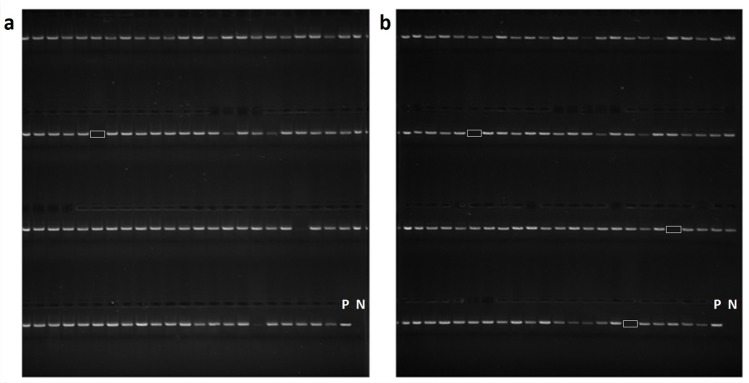
Long-range PCR products and gel electrophoresis. a) Gel electrophoresis of Class I (HLA-A, B, C) using a multiplex primer set. b) Gel electrophoresis of Class II (DQB1 and DRB1) using a multiplex primer set. White box denotes PCR failure. P and N refer to positive control and negative control, respectively.

From October 2014 to October 2015, 10,063 buccal swab samples were successfully typed using this strategy. Among the samples tested during development, the average concentration of DNA isolated from buccal swabs was 3.96 ± 3.74 ng/μL in 100μL volume (n = 96) (**Fig 3**). While this yield was approximately 6-fold lower than DNA isolated from whole blood control specimens (n = 56, 23.99 ± 9.24 ng/μL), the total DNA quantity (range: 1.91–75.5 ng) was sufficient to generate enough amplicons for downstream application after 35 cycles of PCR. Additionally, despite known challenges associated with buccal-derived DNA, robust PCR amplification was achieved on 9,842 NMDP samples (97.8%) during the study period (**Fig 4**), with 221 samples (2.2%) experiencing PCR failure due to low quality and/or quantity of buccal swab DNA.

### NGS typing results

**Fig 5:**
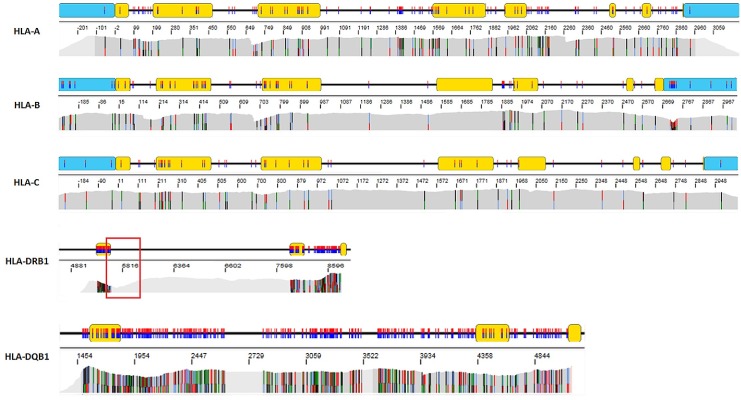
Coverage data taken from a representative sample. The sample was aligned using GenDX Version1.9.0. Blue box, UTR; yellow box, exon. black line, intron. Red box shows the low coverage region in intron 3 of all DRB1 alleles. Uniform coverage was achieved in most loci with the exception of coverage bias found in intron 3 of DRB1 alleles due to the (GT)_x_(GA)_y_ motif.

Overall, > 82% of Illumina MiSeq paired-end sequencing base calls exceeded a quality score of 30 (Q30), indicating that the probability of an incorrect base call was less than 1 in 1000. The average coverage depth was greater than 500 bp across all loci and uniform coverage was generally observed throughout the entire region (**Fig 5**). A slight coverage decrease was found in intron 3 of all DRB1 alleles due to the (GT)_x_(GA)_y_ motif, which was previously reported [11]. Following sequencing, all reads were analyzed using Omixon Twin (V.1.0.7) which combines two independent analysis algorithms (statistical alignment and *de novo* assembly) for robust variant calling. In addition, several embedded quality metrics (read length, read quality, noise ratio, consensus coverage, imbalance ratio, PCR crossover artifact detection, crossmapping reads among different loci and phasing) improved the accuracy and the confidence of allele calling. Among the 9,842 samples (9842*5 loci*2 diploid = 98,420 alleles) that were successfully amplified, 96,686 alleles (96,686/98,420 = 98.2%) were successfully reported by NGS (**Table 1**). When all the quality metrics were met, concordant allele calling was assigned on 95,342 alleles (95,342/96,686 = 98.6%) by both *de novo* assembly and statistical alignment algorithms. Overall, 2,380 alleles (2,380/96,686 = 2.5%) were flagged with QC warning by Omixon Twin that required further manual analysis- 1,344 alleles were reported by NGS after careful manual evaluation (e.g. to ensure complete and sufficient coverage); typing for the remaining 1,036 alleles could not be assigned. Since low DNA quantity and poor sample integrity are known to promote allele dropouts, all homozygous alleles as identified by NGS were confirmed by SSO typing. Parallel SSO testing identified 349 allele dropouts (0.35%) among all buccal swap samples sequenced (**S1 Table**). Allele dropouts were detected across all five loci (A = 111, B = 74, C = 63, DRB1 = 69, and DQB1 = 32). The most common allele dropout was A*02:01:01 which occurred in 26 samples. However, since A*02:01:01 is a high frequency allele, the dropout rate was only 0.57% (26/4571). On the contrary, B*38:01:01 dropout was found in 9 out of 378 alleles, yielding a higher relative dropout rate of 2.38% compared to the A*02:01:01 allele- this suggests a possible primer design issue which requires further optimization. Interestingly, no allele dropouts were detected in 122 blood-based specimens validated in our lab using the same amplification strategy; therefore, primer-independent issues such as DNA quantity/quality were likely significant factors predisposing to allele dropouts in buccal-derived specimens.

**Table 1:** NGS typing results.

9842 samples PCR Amplified(n = 98,420 alleles)		HLA-A	HLA-B	HLA-C	HLA-DRB1	HLA-DQB1	Total
Concordant allelic calling by Omixon Twin		18,622	18,808	19,328	19,110	19,474	95,342
Omixon Twin QC warning	Reported after manual analysis	518	452	118	174	82	1,344
	QC failure	316	364	134	118	104	1,036
Allele Dropout		111	74	63	69	32	349
Total % reported by NGS		97.3%	97.4%	98.7%	98.7%	99.1%	98.2%
Number of NMDP blind QC error(n = 1,302 loci)		0	0	0	2	0	0.16%

NGS typing results were exported from Omixon Twin and reported to the NMDP using Histoimmunogenetics Markup Language (HML) [21]. HML is an electronic messaging format that was developed based on Minimum Information for Reporting Immunogenomic NGS Genotyping (MIRING) reporting guidelines (miring.immunogenomics.org). A MIRING message includes five categories of structured information (message annotation, reference context, full genotype, consensus sequence, novel polymorphism) and three pieces of accessory information (NGS platform documentation, read processing documentation and primary data) [22]. Using this format, HLA genotyping results can be readily updated in accordance with reference and nomenclature databases. During the period of this study, an overall NGS-HLA typing accuracy rate of 99.84% was achieved across all five loci (A, -B, -C, -DRB1, and -DQB1) based on independent testing of 264 blind Quality Control samples by the NMDP (**Table 1**).

### Null, novel and rare alleles detected by NGS in 10,063 buccal swab samples

Unrecognized HLA null alleles or novel alleles may affect the outcome of HSCT [23]. A total of 23 null alleles were identified resulting in a prevalence of 0.023% in this cohort (**Table 2**). The most common null allele detected was C*04:09N. C*04:09N differs from C*04:01:01 in exon 7 where C*04:09N carries a frameshift base pair deletion in codon 341 that leads to a loss of stop codon in exon 8. All C*04:09N alleles detected in this dataset were in linkage with B*44:03.

**Table 2:** Null allele detection by using NGS in 10,063 buccal swab samples.

HLA-allele	Mutation	Numbers detected	Frequency (%)
A*01:16N	Insertion in Exon 3, 532-533insG, in codon 154, causes frameshift and premature stop at codon 196	1	0.005%
A*24:09N	Point mutation at Exon 4, 742-744CAG>TAG, causes Q224X, a premature stop at codon 224	1	0.005%
A*24:11N	Insertion at Exon 4, 627-628insC, in codon 186, causes frameshift and premature stop at codon 196	1	0.005%
C*04:09N	Deletion in Exon 7, 1095delA, in codon 341, causes frameshift and loss of stop codon in exon 8, resulting in the peptide containing an additional 32 amino acids	19	0.094%
C*05:07N	Deletion in Exon 3, 352-353delAC, in codon 94, causes frameshift and premature stop at codon 113	1	0.005%

**Fig 6:**
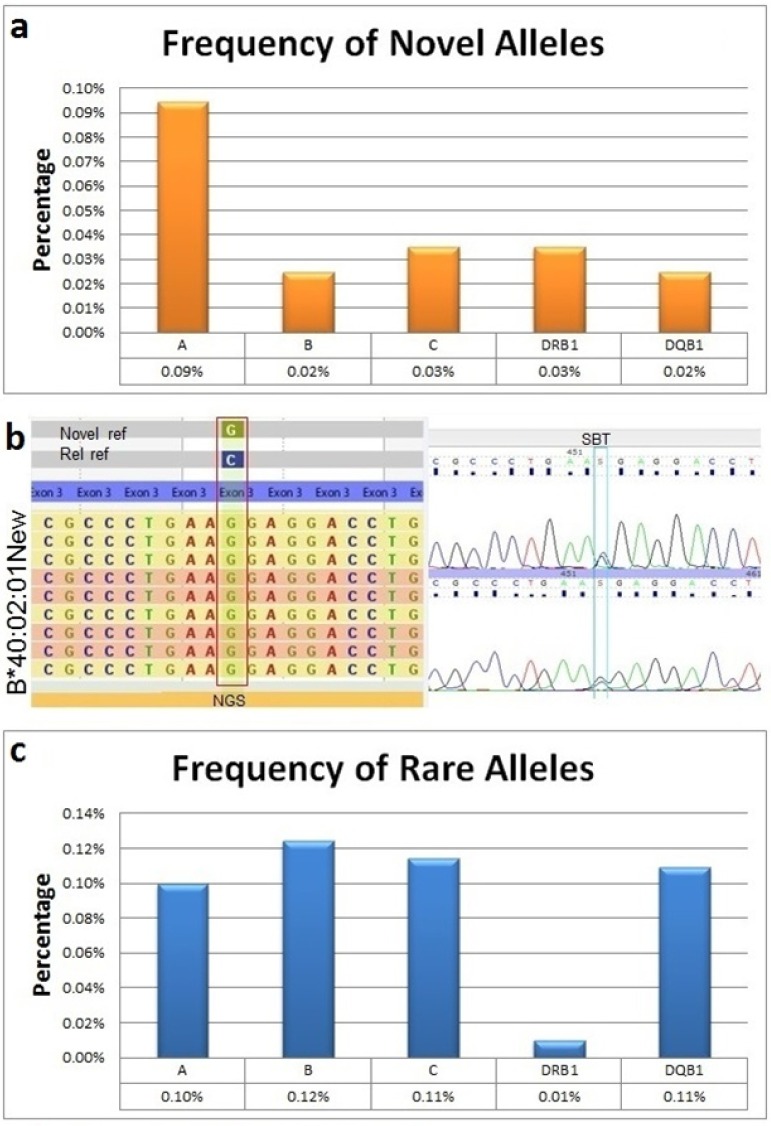
Novel and rare alleles detected by NGS in 10,063 samples. Sequence data was analyzed using Omixon HLA Twin V1.0.7 (3.19.0 IMGT/HLA database). a) Percentage of SBT-confirmed exon novelties shown by HLA locus. b) Example of a novel allele detected by NGS and confirmed using SBT. SBT was unable to determine the cis-trans phase of the exon novelty; in contrast, parallel sequencing by NGS clearly established the phase and location of the novel variant in the B*40:02:01 new allele. c) Percentage of rare alleles detected shown by HLA locus.

The majority of novel polymorphisms detected in this study were found within introns. Due to the paucity of complete intron consensus sequences and a lack of reagents to confirm intron mutations, we elected to prioritize verification of exon novelties, which are known to be the most clinically relevant, and at intron-exon junctions that are predicted to create alternative splicing. During the study period, 42 exon novelties were detected by NGS and confirmed by SBT (**Table 3**). New alleles were identified in 0.09% of HLA-A, 0.02% of -B, and 0.03% of -C alleles (**Fig 6A and 6B**). Since our typing strategy only covered exon 2–4 of class II genes, the percentage of novel alleles detected was probably underestimated. Around 67% of these novelties were non-synonymous mutations (**Table 3**). They were mainly located in exon 1, 2, and 3 of HLA class I (n = 12, 10, and 14 respectively). A*24:02:01NEW was associated with the highest number of mutations, with six detected in exon 1, one in exon 6, and one in exon 2. All novel alleles have been submitted to Genbank and the IMGT/HLA database.

**Table 3:** Exon novelty detection by Omixon Twin 1.0.7 through 10,063 buccal swab samples.

Alleles	Location	Amino Acid change	Number Detected	GenBank Accession#
A*01:01:01NEW	Exon 4, 237 (GGG>AGG)	Non-synonymous	1	KX622665
A*02:01:01NEW	Exon 1, -21 (ATG>GTG)	Non-synonymous	3	KX622669KX622670KX622671
A*11:01:01NEW	Exon 3, 131 (CGC>TGC)	Non-synonymous	1	KX622667
A*11:01:01NEW	Exon 3, 144 (AAG>GAG)	Non-synonymous	1	KX622668
A*24:02:01NEW	Exon 1, -22 (GTC>GTT)	Synonymous	5	KX622659KX622660KX622661KX622662KX622663
A*24:02:01NEW	Exon 1, -19 (CCC>CTC)	Non-synonymous	1	KX622672
A*24:02:01NEW	Exon 2, 76 (GAG>GCG)	Non-synonymous	1	KX622666
A*24:02:01NEW	Exon 6, 316 (AAA>GAA)	Non-synonymous	1	KX622675
A*26:01:01NEW	Exon 1, -7 (GCC>GCT)	Synonymous	1	KX622673
A*30:04:01NEW	Exon 1, -9 (GCC>ACC)	Non-synonymous	1	KX622676
A*33:01:01NEW	Exon 4, 266 (CTC>CTG)	Synonymous	1	KX622664
A*68:01:02NEW	Exon 6, 315 (AGA>GGA)	Non-synonymous	1	KX622674
B*07:02:01NEW	Exon 4, 189 (GTG>ATG)	Non-synonymous	1	KX622688
B*40:02:01NEW	Exon 3, 127 (AAC>AAG)	Non-synonymous	1	KX622685
B*44:02:01NEW	Exon 2, 43 (CCG>CGG)	Non-synonymous	1	KX622686
B*44:34:02NEW	Exon 2, 24 (GCA>TCA)	Non-synonymous	1	KX622684
B*55:01:01NEW	Exon 2, 43 (CCG>TCG)	Non-synonymous	1	KX622687
C*01:02:01NEW	Exon 1, -17 (ACC>AAC)	Non-synonymous	1	KX622683
C*03:04:01NEW	Exon 2, 29 (GAC>GAT)	Synonymous	1	KX622677
C*03:05NEW	Exon 5, 301 (GGA>GAA)	Non-synonymous	1	KX622681
C*04:01:01NEW	Exon 2, 78 (CTG>CTA)	Synonymous	1	KX622679
C*04:01:01NEW	Exon 6, 321 (TGC>TGT)	Synonymous	1	KX622682
C*07:02:01NEW	Exon 3, 163 (ACG>ATG)	Non-synonymous	1	KX622678
C*16:01:01NEW	Exon 4, 229 (GAG>AAG)	Non-synonymous	1	KX622680
DRB1*03:01:01NEW	Exon 3, 149 (CAC>CAG)	Non-synonymous	1	KX622658
DRB1*03:01:01NEW	Exon 3, 133 (CGG>CAG)	Non-synonymous	1	KX622653
DRB1*04:03:01NEW	Exon 2, 43 (GAC>GAT)	Synonymous	1	KX622654
DRB1*07:01:01NEW	Exon 3, 134 (AAC>AAT)	Synonymous	1	KX622652
DRB1*10:01:01NEW	Exon 3, 135 (GGC>AGC)	Non-synonymous	1	KX622656
DRB1*13:01:03NEW	Exon 2, 86 (GTG>GGT)	Non-synonymous	1	KX622655
DRB1*15:01:01NEW	Exon 3, 134 (AAC>AAT)	Synonymous	1	KX622657
DQB1*02:02:01NEW	Exon 3, 118 (TCG>TCA)	Synonymous	1	KX622650
DQB1*03:01:01NEW	Exon 3, 174 (CAC>CAA)	Non-synonymous	1	KX622647
DQB1*03:01:01NEW	Exon 2, 75 (TTG>TTT)	Non-synonymous	1	KX622648
DQB1*05:01:01NEW	Exon 3, 130 (CGG>CAG)	Non-synonymous	1	KX622649
DQB1*05:02:01NEW	Exon 2, 58 (GCC>ACC)	Non-synonymous	1	KX622651

We analyzed our results according to the common and well documented alleles (CWD2.0.0) and identified 92 alleles that were not on the CWD list (**Fig 6C** and **Table 4**). Interestingly, B*15:220 was detected in 11 of the 10,063 samples with an allele frequency of 0.055%. B*15:220 differs from B*15:03:01 by one nucleotide in exon 1 where B*15:03:01 carries a cytosine (C) and B*15:220 carries a thymine (T). The 47C->T substitution results in an amino acid change from alanine to valine in the signal peptide at amino acid position −9. Other rare alleles included C*04:12 and DQB1*06:01:03, both detected 3 times in the cohort (frequency = 0.015%). These alleles should be re-characterized as common and well defined alleles.

**Table 4:** Rare alleles detected by NGS that are not present on CWD 2.0.0 in 10,063 samples.

HLA-allele	G group	Numbers detected	Allele Frequency (%)
A*01:23		1	0.005%
A*01:37	A*01:01:01G	2	0.010%
A*02:37		1	0.005%
A*02:66	A*02:01:01G	1	0.005%
A*02:110		1	0.005%
A*03:102		1	0.005%
A*02:194		1	0.005%
A*11:47		1	0.005%
A*11:108	A*11:01:01G	1	0.005%
A*23:18	A*23:01:01G	1	0.005%
A*24:46		1	0.005%
A*26:30		1	0.005%
A*30:24	A*30:01:01G	1	0.005%
A*30:28		1	0.005%
A*66:17	A*66:01:01G	1	0.005%
A*68:44		1	0.005%
A*01:16N		1	0.005%
A*68:67		1	0.005%
A*68:93		1	0.005%
B*07:24		2	0.010%
B*07:29		1	0.005%
B*15:146	B*15:01:01G	1	0.010%
B*18:15		1	0.005%
B*18:28		1	0.005%
B*18:34		1	0.005%
B*15:220	B*15:03:01G	11	0.055%
B*27:13	B*27:05:02G	1	0.005%
B*27:25		1	0.005%
B*49:16		1	0.005%
B*58:11	B*58:01:01G	1	0.005%
B*40:126		1	0.005%
B*40:80		1	0.005%
B*44:102		1	0.005%
C*02:15		1	0.005%
C*02:21		1	0.005%
C*02:56		1	0.005%
C*03:21		1	0.005%
C*03:87		1	0.005%
C*04:12		3	0.015%
C*04:40		1	0.005%
C*04:46		1	0.005%
C*03:129		2	0.010%
C*05:37	C*05:01:01G	1	0.005%
C*06:10		1	0.005%
C*07:23		1	0.005%
C*07:74	C*07:02:01G	1	0.005%
C*07:85		1	0.005%
C*07:179		1	0.005%
C*12:06		1	0.005%
C*12:07		1	0.005%
C*12:24		1	0.005%
C*15:24		1	0.005%
C*16:14		1	0.005%
DRB1*03:19		1	0.005%
DRB1*03:49		1	0.005%
DQB1*02:07		1	0.005%
DQB1*02:10	DQB1*02:01:01G	1	0.005%
DQB1*02:26		2	0.010%
DQB1*02:28		1	0.005%
DQB1*03:03:04	DQB1*03:03:02G	1	0.005%
DQB1*03:24	DQB1*03:01:01G	2	0.010%
DQB1*03:25		1	0.005%
DQB1*03:55		1	0.005%
DQB1*03:58		1	0.005%
DQB1*05:02:07	DQB1*05:02:01G	1	0.005%
DQB1*06:01:03	DQB1*06:01:01G	3	0.015%
DQB1*06:01:08		1	0.005%
DQB1*06:02:02		1	0.005%
DQB1*06:07:01		1	0.005%
DQB1*06:14:01		1	0.005%
DQB1*06:16		2	0.010%
DQB1*06:50		1	0.005%

## Discussion

To date, several groups have developed different protocols and workflows for HLA genotyping by NGS; however, most of the published studies are small in sample size [9,10,12–15,18,19] and the majority were performed using blood-based specimens. Here, we report our NGS-HLA typing experience performed on buccal DNA isolated from NMDP registry donors. Of the 10,063 samples tested over the one-year period, robust PCR amplification was accomplished in 9,842 samples (97.8%). Overall, a typing accuracy of 99.8% was achieved based on independent testing of 265 blind QC samples embedded into the study cohort. A total of 42 exon novelties, 23 null alleles, and 92 alleles were identified in this study.

There are many advantages to using buccal swab specimens for clinical testing. While peripheral blood is routinely used for PCR-based assays, blood sampling is invasive, time-consuming, and expensive. These limitations are challenging for high volume donor recruitment typing, particularly when participants are dispersed geographically. Buccal swabs, on the other hand, are considered a convenient alternative for collecting genetic material, as they are relatively inexpensive, noninvasive and can be mailed out for self-collection and return-mailed for centralized testing [24,25]. In our study, DNA yield from buccal swabs was approximately six times lower than DNA isolated from blood samples. DNA degradation can occur when bacterial contamination digests DNA strands. Therefore, keeping buccal swabs in a cool and dry environment can mitigate the degradation process by limiting the generation of inhibitory substances. The variation of the DNA yield is also influenced by individual collection techniques. Samples with low DNA copy number are known to be more subject to PCR inhibitory substances. Despite these known challenges, robust long range PCR was achieved on 97.8% samples in our study, with a high percentage of specimens (98.2%) being reported successfully downstream.

One problem associated with PCR of diploid genomic DNA is the preferential amplification of heterozygous alleles. This manifests as allele imbalance in downstream NGS analysis- depending on the threshold depth used for genotype calling, the analysis software may inappropriately perceive the low-coverage heterozygous allele as background “noise”, leading to assignment of homozygosity with the preferentially amplified allele [26,27]. To characterize this phenomenon, we performed parallel SSO typing of all homozygous alleles assigned by NGS in this study. Overall, an allele dropout rate of 0.35% was observed based on confirmatory typing of 6,790 homozygous alleles (A = 2,220, B = 972, C = 1,470, DRB1 = 650, DQB1 = 1,478). Preferential amplification may not account for all of the allele dropouts identified in this cohort. It is well known that PCR bias is subject to variations in DNA quantity or quality, presence of PCR inhibitors, variations in pipetting volumes of reagents, imprecisions in thermocycler temperatures and human errors. This type of bias is usually random and not reproducible. Repeat testing of the sample may resolve the problem. Alternatively, nonrandom amplification failures can result from extreme GC content, and preferential polymerase recognition sites [28]. Additionally, allele dropout can be caused by sequence specific variations; in this case, amplification failures occur when a given primer cannot stably hybridize to its specific complementary sequence binding site, or when novel mutations are found in the primer binding site. This type of dropout requires redesign of primers or alternatively using two different set of primers to capture the same allele.

Currently, only 3.21% of HLA-A (102 alleles), 4.60% of HLA-B (182 alleles), 7.71% of HLA-C (211 alleles) and 1.42% of HLA-DRB1 (25 alleles) have complete nucleotide sequences from 5’-UTR to 3’-UTR [29]. The typing strategy described here utilizes long-range PCR to amplify full-length sequences of class I genes (HLA-A, B, C), and uninterrupted sequences from exon 2 to beginning of exon 4 of class II (HLA-DRB1 and DQB1), thereby providing valuable intron information involving previously unmapped regions. Intron sequences constitute approximately 30% of the human genome [30]; however, the function of introns is not completely understood. Recent NGS studies have shown that noncoding RNAs (ncRNAs), including miRNA, siRNA, long noncoding RNAs (lncRNAs) and small nucleolar RNAs (snoRNAs) found in intron regions of the human genome may play an important role in regulating gene expression [31,32]. The role of intron sequences in HLA expression and their importance in HSCT have recently been demonstrated in HLA-C and DP. HLA-C is expressed at a level approximately 10-fold lower than HLA-A and B antigens [33]. The mechanism underlying this varied expression was unknown until recently when Kulkarni and colleagues demonstrated differential regulation of HLA-C expression through miRNA. miRNA is known to bind to specific sites in the 3’ UTR, resulting in post-transcriptional repression, cleavage or destabilization [34]. Kulkarni and colleagues showed that a single polymorphism (SNP) in the 3’ UTR of HLA-C affects binding of the has-miR-148 miRNA to its target site, resulting in a relatively low HLA-C surface expression; in contrast, alleles without has-miR-148 binding escape post-transcriptional regulations and have high HLA-C expression [35]. In HSCT, increased expression of the recipient’s mismatched HLA-C is associated with an increased risk of grade III and grade IV graft-versus-host disease (GVHD), non-relapse mortality and overall mortality [36]. In addition, a SNP in the 3’ UTR of HLA-DP has been reported to alter HLA-DP expression [37]. Position rs9277534A is associated with low DP expression while rs9277534G is associated with high expression. In HSCT, the risk of acute GVHD was higher for recipients with rs9277534G-linked HLA-DPB1 mismatches than for recipients with rs9277534A-linked HLA-DPB1 [37]. Intronic mutations can also result in null allele expression. Although we did not identify intronic null mutations in our dataset, there are seven null alleles in the IMGT/HLA database (v.3.23.0) that lead to null protein expression due to alterations in mRNA splicing. Since the serological method is no longer commonly used for HLA typing, aberrant HLA expression due to unrecognized mutations cannot be fully appreciated until complete gene sequences are routinely interrogated and curated.

Herein, we report our experience of high-throughput, high-resolution NGS-HLA typing for NMDP donors with high accuracy. The combination of automation and robust data analysis software makes NGS typing a cost effective solution for sequencing registry donors. Further, the ability to deliver unambiguous typing holds great promise to facilitate timely donor identification for HSCT recipients. Since >90% of HLA class I and class II genes do not have complete sequences [29], our results made it possible to fill in gaps in the existing HLA database. By analyzing polymorphisms in the entire region of HLA genes, the importance of polymorphisms outside ARS in introns, promoters, enhancers, and UTRs can be further revealed in transplantation, autoimmunity, diseases association, and pharmacogenomics.

## Supporting Information

S1 Table Allele dropout detection in 10,063 buccal swab samples. (DOCX)
