# Supplementary material for: Application of High-Throughput Next-Generation Sequencing for HLA Typing on Buccal Extracted DNA: Results from over 10,000 Donor Recruitment Samples
Source: PLoS One. 2016 Oct 31;11(10):e0165810. doi: 10.1371/journal.pone.0165810 (PMC5087893; doi:10.1371/journal.pone.0165810)
Supplement: S1 Table — (DOCX) [file pone.0165810.s001.docx]

**S1 Table. Allele dropout detection in 10,063 buccal swab samples**

| **A Dropout** | **Number** | **B Dropout** | **Number** | **C Dropout** | **Number** | **DRB1 Dropout** | **Number** | **DQB1 Dropout** | **Number** |
| --- | --- | --- | --- | --- | --- | --- | --- | --- | --- |
| A*01:01:01 | 9 | B*07:02:01 | 9 | C*01:02:01 | 5 | DRB1*04:01:01 | 14 | DQB1*02:01:01 | 9 |
| A*02:01:01 | 26 | B*07:05:01 | 2 | C*02:02:01 | 2 | DRB1*04:02:01 | 4 | DQB1*02:02:01 | 6 |
| A*02:02 | 1 | B*08:01:01 | 3 | C*02:02:02 | 2 | DRB1*04:03:01 | 6 | DQB1*03:01:01 | 5 |
| A*02:07:01 | 2 | B*14:01:01 | 1 | C*02:10 | 4 | DRB1*04:04:01 | 13 | DQB1*03:02:01 | 1 |
| A*02:17:01 | 1 | B*14:02:01 | 3 | C*03:03:01 | 4 | DRB1*04:05:01 | 3 | DQB1*04:01:01 | 1 |
| A*03:01:01 | 15 | B*15:01:01 | 1 | C*03:04:01 | 7 | DRB1*04:07:01 | 3 | DQB1*05:01:01 | 2 |
| A*11:01:01 | 4 | B*15:04:01 | 1 | C*03:04:02 | 1 | DRB1*04:08:01 | 3 | DQB1*05:03:01 | 2 |
| A*23:01:01 | 5 | B*15:10:01 | 1 | C*04:01:01 | 4 | DRB1*04:11:01 | 3 | DQB1*05:05 | 1 |
| A*24:02:01 | 9 | B*18:01:01 | 2 | C*04:07 | 1 | DRB1*07:01:01 | 1 | DQB1*06:01:01 | 1 |
| A*24:03:01 | 1 | B*27:05:02 | 1 | C*05:01:01 | 5 | DRB1*08:01:01 | 1 | DQB1*06:02 | 4 |
| A*25:01:01 | 1 | B*35:01:01 | 2 | C*06:02:01 | 2 | DRB1*11:01:01 | 1 |  |  |
| A*26:01:01 | 7 | B*35:03:01 | 2 | C*07:01:01 | 9 | DRB1*11:15 | 1 |  |  |
| A*26:08 | 1 | B*37:01:01 | 1 | C*07:02:01 | 6 | DRB1*13:01:01 | 3 |  |  |
| A*29:01:01 | 1 | B*38:01:01 | 9 | C*07:04:01 | 1 | DRB1*14:04:01 | 1 |  |  |
| A*29:02:01 | 7 | B*39:02:01 | 1 | C*08:01:01 | 1 | DRB1*14:09 | 1 |  |  |
| A*30:02:01 | 2 | B*39:06:01 | 2 | C*08:02:01 | 2 | DRB1*15:01:01 | 8 |  |  |
| A*30:10 | 1 | B*39:10:01 | 2 | C*12:03:01 | 2 | DRB1*15:03:01 | 2 |  |  |
| A*31:01:01 | 3 | B*39:24:01 | 1 | C*15:02:01 | 1 | DRB1*16:03 | 1 |  |  |
| A*33:01:01 | 3 | B*40:01:01 | 1 | C*16:01:01 | 3 |  |  |  |  |
| A*34:01:01 | 1 | B*40:02:01 | 3 | C*17:01:01 | 1 |  |  |  |  |
| A*66:02 | 2 | B*40:27:01 | 1 |  |  |  |  |  |  |
| A*68:01:01 | 6 | B*41:01:01 | 1 |  |  |  |  |  |  |
| A*68:02:01 | 3 | B*44:02:01 | 3 |  |  |  |  |  |  |
|  |  | B*44:03:01 | 1 |  |  |  |  |  |  |
|  |  | B*44:04 | 1 |  |  |  |  |  |  |
|  |  | B*45:01:01 | 1 |  |  |  |  |  |  |
|  |  | B*46:01:01 | 1 |  |  |  |  |  |  |
|  |  | B*48:03:01 | 1 |  |  |  |  |  |  |
|  |  | B*49:01:01 | 3 |  |  |  |  |  |  |
|  |  | B*50:01:01 | 2 |  |  |  |  |  |  |
|  |  | B*51:01:01 | 2 |  |  |  |  |  |  |
|  |  | B*55:01:01 | 1 |  |  |  |  |  |  |
|  |  | B*56:01:01 | 2 |  |  |  |  |  |  |
|  |  | B*57:01:01 | 3 |  |  |  |  |  |  |
|  |  | B*58:01:01 | 3 |  |  |  |  |  |  |
|  |  |  |  |  |  |  |  |  |  |
|  |  |  |  |  |  |  |  |  |  |
|  |  |  |  |  |  |  |  |  |  |
